# The Role of Serum Levels of Neurofilament Light (NfL) Chain as a Biomarker in Friedreich Ataxia

**DOI:** 10.3389/fnins.2021.653241

**Published:** 2021-03-02

**Authors:** Bernice Frempong, Robert B. Wilson, Kimberly Schadt, David R. Lynch

**Affiliations:** ^1^Division of Neurology, Children's Hospital of Philadelphia, Philadelphia, PA, United States; ^2^Departments of Neurology and Pediatrics, University of Pennsylvania, Philadelphia, PA, United States; ^3^Department of Pathology and Laboratory Medicine, Children's Hospital of Philadelphia, Philadelphia, PA, United States

**Keywords:** Ataxia, biomarker, neurodegenerative, clinical trial, axon

## Introduction

Friedreich Ataxia (FRDA) is a progressive neurological and systemic disorder that affects about one in 50,000 people worldwide (Strawser et al., 2017). It is caused by mutations, usually GAA repeat expansions (96%) but also point mutations or deletions (4%), in the *FXN* gene, resulting in decreased production of functional frataxin protein (Babady et al., 2007; Delatycki and Bidichandani, 2019). GAA length on the shorter allele inversely correlates with disease severity (Strawser et al., 2017). Frataxin is a small mitochondrial protein that functions in iron-sulfur-cluster biosynthesis (Colin et al., 2013). Its deficiency leads to difficulties in production of cellular ATP as well as sensitivity to reactive oxygen species *in vitro* (Rötig et al., 1997; Lodi et al., 2001; Pastore and Puccio, 2013; DeBrosse et al., 2016). These properties lead to neurological injury and clinical impairment, including ataxia, dysarthria, sensory loss, and weakness in FRDA patients. While most literature has focused on neurodegeneration in FRDA, the disorder also has a large developmental component (Koeppen et al., 2017a,b). In addition, individuals with FRDA develop cardiomyopathy, scoliosis and sometimes diabetes mellitus. The cardiomyopathy of FRDA is characterized by early hypertrophy, with later progression to fibrosis and systolic dysfunction, leading to death from end-stage heart failure (Tsou et al., 2011; Lynch et al., 2012; Strawser et al., 2017). Many agents are in development for FRDA, including some designed to ameliorate mitochondrial dysfunction and others that seek to increase levels of functional frataxin (Strawser et al., 2014; Li et al., 2015; Lynch et al., 2018, 2021; Piguet et al., 2018; Zesiewicz et al., 2018a,b; Belbella et al., 2019; Rodríguez-Pascau et al., 2021).

## NfL as a Biomarker of FRDA and Other Diseases

In many neurodegenerative diseases, the need for disease-modifying treatments is facilitated by identification of biomarkers to track disease progression. Such markers can capture subclinical changes in a rapid manner and show evidence of target engagement in clinical trials in slowly progressive neurological disorders. In other neurological disorders, including Multiple Sclerosis (MS), Alzheimer's disease (AD), and Parkinson's disease (PD), neurofilament light chain levels (NfL) in body fluids such as serum, plasma or CSF may provide a biomarker for tracking disease activity including progression (Bridel et al., 2019; Forgrave et al., 2019; Aktas et al., 2020; Del Prete et al., 2020; Milo et al., 2020; Thebault et al., 2020; Wang et al., 2020). Neurofilaments are cytoskeletal proteins located in both the peripheral and central nervous system, particularly in larger myelinated axons. They play a significant role in axonal growth and the determination of axonal caliber (Hsieh et al., 1994; Kurochkina et al., 2018; Bott and Winckler, 2020). Logically, as axons are damaged and die in neurodegenerative processes, NfL should leak into the interstitial space, then into CSF and plasma. Thus, concentrations of NfL should generally increase as neurodegenerative diseases progress and should reflect disease activity. For example, in progressive MS, NfL appears to track with neuronal and axonal death, the stage of disease, and treatment response. NfL concentration in the CSF of MS patients parallels T2 lesion changes on MRI. Similarly, plasma NfL concentration is higher in AD patients than in controls and is associated with greater cognitive deficit. Such findings suggest that NfL is a promising biomarker for determining the stage of disease, tracking progression and aiding in identification of disease-modifying treatments in neurological disorders. However, in stable MS, NfL levels may not track with clinical dysfunction, providing a reminder that changes in biomarkers must be interpreted in the context of clinical changes (Aktas et al., 2020).

In FRDA, data on NfL levels in serum is more complex. Most features of FRDA depend on genetic severity (GAA repeat length) and worsen over the course of time, thus correlating positively with disease duration or age (Strawser et al., 2017). Overall, the two main determinants of clinical severity in FRDA are genetic severity and disease duration. In the three studies evaluating serum NfL in FRDA patients, serum NfL is elevated in patients with FRDA when compared to controls and carriers (Zeitlberger et al., 2018; Clay et al., 2020; Hayer et al., 2020). This shows that serum NfL levels reflect a pathological process in FRDA. However, in these three studies NfL levels generally do not correlate with markers of clinical or genetic severity. Moreover, while NfL levels correlate positively with age in non-FRDA patients (controls and carriers) in cross sectional analysis, in FRDA patients, NfL levels are highest in young children and *decrease* with age as the disease progresses (Clay et al., 2020). Thus, serum NfL is paradoxically high in young individuals and far lower in older individuals with more severe disease. At later ages it even overlaps with control values. A greater genetic severity in early onset individuals could explain some of this paradox; however, NfL levels overall do not correlate with GAA repeat length after accounting for age, and they even appear to correlate inversely with GAA length in some of these studies. Accounting for age, individuals who are more severe genetically have lower levels of NfL. Interestingly, Nfl levels are relatively stable over 1–2 years; consequently, NfL levels could be used as an assessment of therapeutic response over the time used in most clinical trials. Still, while NfL may provide a biomarker of FRDA in some manner, the relationship of NfL to disease progression is complex suggesting its utility may be limited to certain situations.

## Discussion

**Figure 1:**
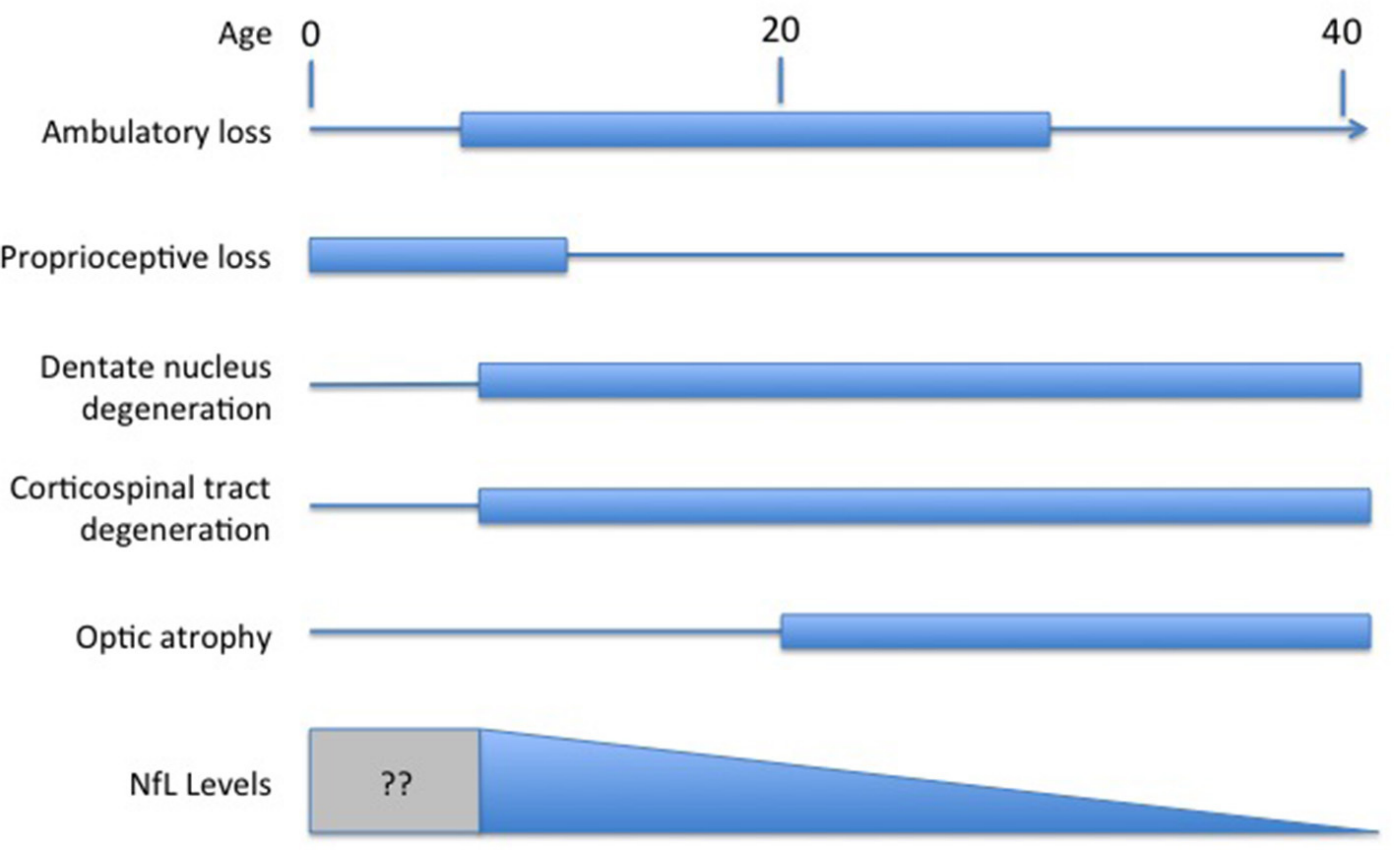
Temporal course of changes in FRDA. Diagram illustrating the contrasting temporal course of clinical changes in FRDA along with serum NfL levels over time (amalgamated from Bridel et al., 2019; Del Prete et al., 2020; Wang et al., 2020). Clinical changes are presented based on the clinical course of an early onset individual (onset between ages 5−10). At present, no study has measured NfL in the presymptomatic period before age five.

Understanding the exact meaning of NfL levels in serum and how they reflect disease activity in FRDA would facilitate their use as a marker of FRDA. In most other disorders, NfL is viewed as a marker of neurodegeneration of either axons or other neuronal regions. Degeneration in FRDA, though, is complex, including both peripheral nerve degeneration (including very early degeneration of proprioceptive afferents) with later degeneration of central nervous system axons; loss of central nervous system elements controls most of the clinical progression of the disease (Selvadurai et al., 2016; Koeppen et al., 2017a; Strawser et al., 2017; Marty et al., 2019; Rezende et al., 2019; Harding et al., 2020; Naeije et al., 2020). Brain imaging studies are typically normal early in disease, with the exception of atrophy of the cervical spinal cord, with progressive loss of CNS pathways later (Selvadurai et al., 2016; Koeppen et al., 2017b; Marty et al., 2019; Rezende et al., 2019; Harding et al., 2020; Naeije et al., 2020). Thus, serum NfL levels in FRDA, with high values early in disease, are discrepant from the tangible loss of CNS axons by MRI and the loss of specific functional clinical systems (Figure 1). The present data on serum NfL levels could be explained by a relatively large early loss of peripheral axons that does not contribute to clinical progression. Similarly, the inverse correlation with GAA repeat length in early disease might lead to a large developmental deficit at presentation. This in turn might lead to lower serum NfL levels during neurodegeneration. This interpretation would be consistent with the prevailing concept of NfL levels reflecting a relatively passive leakage from dying neurons into surrounding fluids and eventually to the serum.

Alternatively, increased levels of NfL could reflect other components of the pathophysiology of FRDA in a manner not directly associated with cell death. FRDA is associated with abnormalities in lipid metabolism as well as lipid peroxidation (Navarro et al., 2010; Obis et al., 2014; Abeti et al., 2016; Chen et al., 2016; Tamarit et al., 2016; Cotticelli et al., 2019; Turchi et al., 2020). Both could lead to membranes that are inherently more permeable than normal, with consequent loss of NfL from the cell. Why these processes would decrease with age, however, is unclear.

Still, other processes might contribute to the paradox of elevated NfL levels early in FRDA. NfL levels must to some degree reflect its synthesis, as increased synthesis leads to higher levels of soluble NfL (before it is incorporated into neurofilaments) that should more readily efflux from neurons cells than NfL assembled into intact neurofilaments. Increased synthesis of structural proteins in axons occurs in response to injury and during neuronal regeneration (Pearson et al., 1988; Havton and Kellerth, 2001; Toth et al., 2008; Balaratnasingam et al., 2011; Yin et al., 2014; Liu et al., 2016), and neurofilaments play different roles in development than simply structural maintenance. The very high levels of NfL early in FRDA could reflect attempts at regeneration that become more impaired as the disease progresses, leading to falling levels of NfL later in the course of the disorder. In general, the plasticity of the nervous system decreases with aging, matching the falls in serum NfL over time in FRDA (Bouchard and Villeda, 2015). Thus, elevated levels of serum NfL early in the course of FRDA could be driven by enhanced synthesis of NFL during regeneration superimposed on increased membrane fragility.

Interestingly, cardiac troponin levels are elevated in FRDA serum during the period of hypertrophic disease, long before cardiomyocytes die and cardiac fibrosis develops (Friedman et al., 2013; Plehn et al., 2018; Legrand et al., 2020). Such elevated levels of troponin in early FRDA cardiomyopathy might result from similar mechanisms to the elevated levels of NfL early in FRDA (Thebault et al., 2020).

A final possibility is that both cell-loss and cell-repair mechanisms—and possibly still other mechanisms—mediate the changes in NfL in FRDA. Such interpretations may only be distinguishable over time with collection of long-term serial data, and with collection of data during the presymptomatic period. Furthermore, a more complete characterization of the features of immunoreactive NfL in FRDA serum may be helpful. While the assays used are specific for NfL, they do not assess whether it represents full-length protein. This does not change the observation that Nfl levels can serve as biomarkers of disease in FRDA. However, without understanding the reason for the unusual distribution of NfL values, it is difficult to provide precise interpretations for clinical trial results. Normalization of biomarker levels can provide evidence for benefit in the correct circumstances, but can also reflect impairment of compensatory mechanisms, thus being associated with deleterious effects. A deeper understanding of the mechanisms of NfL elevation in serum in FRDA is needed to make it a useful biomarker in FRDA.

## Author Contributions

BF created the first draft and edited the final work. RW and KS provided ideas, editing, and critical review. DL contributed to the first draft, performed critical review, ideas for the project, and designed the figure. All authors contributed to the article and approved the submitted version.

## Conflict of Interest

The authors declare that the research was conducted in the absence of any commercial or financial relationships that could be construed as a potential conflict of interest.

## Abbreviations

FRDA: Friedreich Ataxia
NfL: Neurofilament light chain
GAA: Guanine-adenine-adenine
MS: Multiple sclerosis
PD: Parkinson's disease
CSF: Cerebrospinal fluid
AD: Alzheimer's disease
CNS: central nervous system.
